# Rituximab in steroid-refractory immune-related pancreatitis: a case report

**DOI:** 10.3389/fonc.2023.1205720

**Published:** 2023-07-31

**Authors:** Armando Santoro, Silvia Masini, Raffaele Cavina, Maria Chiara Tronconi, Fabio De Vincenzo

**Affiliations:** ^1^ Department of Biomedical Sciences, Humanitas University, Milan, Italy; ^2^ IRCCS Humanitas Research Hospital, Humanitas Cancer Center, Milan, Italy

**Keywords:** rituximab, steroid-refractory pancreatitis, immune-related adverse event, atezolizumab, immune checkpoint inhibitors

## Abstract

The use of immune checkpoint inhibitors (ICIs) for treating several types of cancer is increasing, but they may be associated with immune-related adverse events (irAEs). Pancreatitis is a rare irAE, mostly responsive to steroid treatment. There are no published data on the management of steroid-refractory ICI-induced pancreatitis. Rituximab has shown efficacy in the setting of relapsing non-ICI-induced autoimmune pancreatitis. However, its use has not been tested for treating immunotherapy-related pancreatitis. Here, we present the case of a patient with steroid-refractory immune-related pancreatitis successfully treated with rituximab as a potential strategy for irAE management.

## 1 Introduction

The use of immune checkpoint inhibitors (ICIs), including cytotoxic T-lymphocyte-associated protein 4 (CTLA4)-directed and programmed cell death protein 1 (PD-1) and its ligand 1 (PD-L1)-targeting monoclonal antibodies, is increasing thanks to the efficacy shown in treating various cancer types (1). However, their use can lead to immune-related adverse events (irAEs), resulting from excessive self-immune responses against normal organs (1–3). ICI-related toxicity incidence and prevalence remain unclear. The most common and well-documented irAEs are gastrointestinal, dermatological, pulmonary, and endocrine toxicities (1–5). A less common irAE is represented by pancreatic toxicity reported in 0.9%–3% with anti-CTLA-4, 0.5%–1.6% with anti-PD-L1, and 1.2%–2.1% with combined anti-CTLA-4 and anti-PD-L1, respectively (6). The clinical presentation varies from asymptomatic amylase and/or lipase elevations to, more rarely, acute pancreatitis (7–10). Two meta-analyses reported a higher risk of pancreatic toxicity with ICIs than with chemotherapy or placebo. However, both studies were limited by the lack of clinical and radiologic data (11, 12). The National Comprehensive Cancer Network and the American Society of Clinical Oncology developed therapeutic algorithms to aid the diagnosis and treatment of these adverse events, consisting of corticosteroids for the standard treatment of most high-grade irAEs (4, 13). However, only the National Comprehensive Cancer Network guidelines reported indications for the first-line management of immune-related pancreatitis but not for steroid-refractory patients.

On the other hand, according to the international consensus for the treatment of non-ICI-induced autoimmune pancreatitis (AIP), steroid treatment is the first line for remission induction in patients with active untreated AIP (14). However, when glucocorticoid monotherapy fails to induce remission or control the disease, rituximab is suggested as a second-line treatment. In particular, the Mayo Clinic Experience reported a complete remission rate of 83% with rituximab in a series of patients with steroid-resistant AIP; furthermore, no relapses were observed while on maintenance therapy (15).

In the present case report, a 53-year-old woman was diagnosed with atypical metastatic carcinoid of the lung in November 2020. Staging by whole-body computed tomography (CT) scan and 18F-fluorodeoxyglucose positron emission tomography (18F-FDG-PET) showed bilateral involvement of the lungs and adrenal glands and multiple lesions in the thyroid gland. An ultrasound scan displayed suspicious nodules in the scalp and right parotid gland. PET with gallium-68 was negative. The diagnosis was carried out with a transthoracic biopsy. Histological examination revealed Ki67 at 30% and PD-L1 at 30%. Fine-needle aspiration confirmed thyroid metastasis. Given the histopathological characteristics, the aggressive onset, and the disease behavior, we decided to treat our patient with six 21-day courses of carboplatin (area under the curve of 5 mg/ml/min) and etoposide (100 mg/mq) combined with atezolizumab (1,200 mg) from December 2020 to April 2021 (16). Two weeks after the last cycle of induction chemotherapy, the patient presented to the emergency department with grade 3 (G3) abdominal pain, elevated serum lipase and amylase levels (both G1), and inhomogeneous pancreas on an abdominal ultrasound scan.

Here, we report on rituximab treatment to address the patient’s steroid-refractory and relapsing ICI-related pancreatitis. This is the first reported case of a patient receiving rituximab with a positive clinical outcome.

## 2 Case presentation

**Figure 1:**
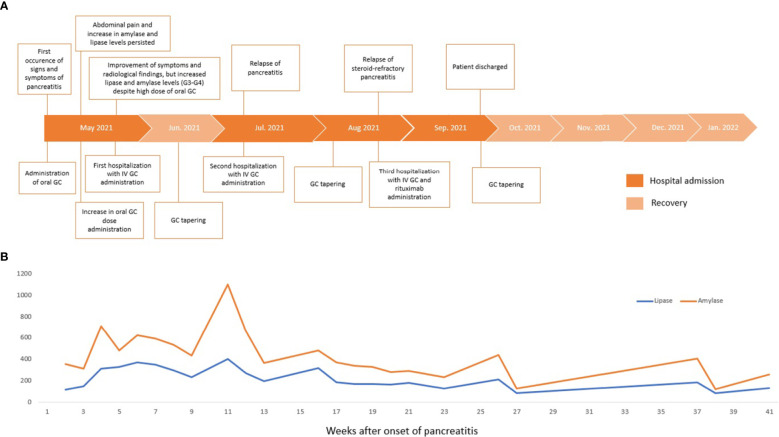
Timeline of clinical presentation, interventions, and outcomes **(A)**. Serum lipase and amylase levels trend during clinical management **(B)**. GC, glucocorticoids; IV, intravenous.

**Figure 2:**
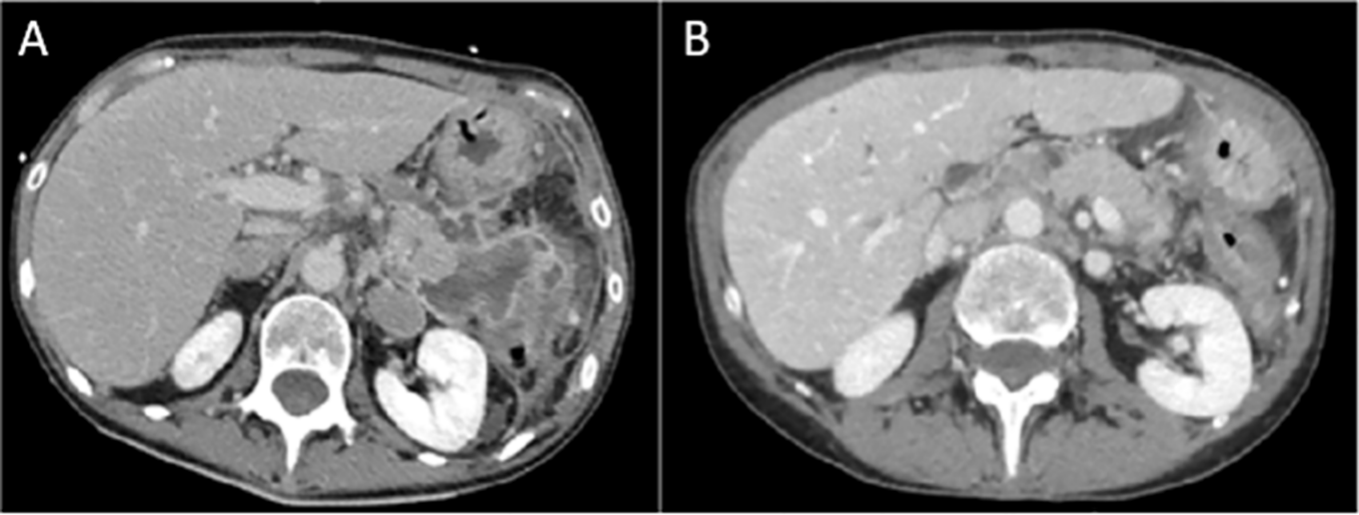
The baseline abdominal CT scan **(A)** shows findings of pancreatitis and a multiloculated fluid and hemorrhagic collection in the pancreatic tail, with a maximum size of 6.5 cm × 5 cm. After 16 weeks since the last dose of rituximab, CT scan **(B)** shows complete resolution of pancreatitis, with homogeneous pancreatic parenchyma and fluid collection.

The clinical events and the type and duration of interventions from the emergency department admission onward are reported in **Table 1** and **Figure 1**. Atezolizumab was permanently discontinued, and oral administration of prednisone at 0.75 mg/kg daily was given for 7 days. On subsequent re-evaluation, the patient’s symptoms and pancreatic enzyme elevation persisted despite continued treatment with analgesics and steroids. The CT scan revealed imaging features of pancreatitis. Therefore, steroid treatment was increased to 1 mg/kg/day. The patient reported pain improvement 2 weeks later, but concomitantly G3–G4 elevated serum amylase and lipase levels were detected. A new CT scan showed the first signs of pancreatitis resolution compared with the previous scan. The patient was hospitalized, and treatment was delivered, including fasting, parenteral nutrition, steroid agents (methylprednisolone 1 mg/kg intravenous twice a day), and opioids for pain management, with clinical benefit. At discharge, oral prednisone at 1.5 mg/kg/day was started and tapered. After 2 months, despite maintenance treatment with glucocorticoids (0.5 mg/kg/day), the patient presented again to the emergency department with severe abdominal pain and increased G3–G4 amylase and lipase, requiring hospitalization. A gastroenterologist monitored the patient during admission. A CT scan evaluation confirmed radiologic features of pancreatitis. MRI and magnetic resonance cholangiopancreatography identified a fluid collection in the pancreatic tail involving the spleen associated with restricted diffusion-weighted imaging and diffuse stenosis of the main pancreatic duct, suggestive of AIP (17, 18). The same steroid regimen was established, but this time steroid responsiveness was not as expected, as the radiologic findings of pancreatitis on CT improved only slightly. Therefore, the patient was discharged with high-dose oral steroid therapy (prednisone at 2 mg/kg/day). During this hospitalization, new-onset diabetes was diagnosed, and insulin was initiated. After 2 weeks from the start of steroid tapering, there was a relapse with severe abdominal pain and elevation of serum pancreatic markers, together with C-reactive protein levels. A CT scan showed worsening of the known pancreatic fluid collection with hemorrhagic development (**Figure 2**). In the course of this third hospital admission, we started a new regimen made of piperacillin/tazobactam, methylprednisolone, and morphine intravenous to achieve pain control and treat undergoing covered infections. Abdominal MRI was repeated, describing several new fluids and hemorrhagic collections with far worse stenosis of the main pancreatic duct, confirming the poor responsiveness to steroid treatment associated with clinical deterioration; no increase in carbohydrate antigen 19-9 (CA 19-9) was observed.

**Table 1:** Therapy administration schedule.

Clinical event	Medical intervention	Treatment duration (days)
First symptoms	Oral prednisone at 0.75 mg/kg/day	7
First follow-up	Oral prednisone at 1 mg/kg/day	14
First hospitalization	IV methylprednisolone at 2 mg/kg/day	5
First discharge	Oral prednisone at 1.5 mg/kg/day	5
	Oral prednisone at 1 mg/kg/day	5
	Oral prednisone at 0.5 mg/kg/day	42
Second hospitalization	IV methylprednisolone at 2 mg/kg/day	18
Second discharge	Oral prednisone at 2 mg/kg/day	7
	Oral prednisone at 1.5 mg/kg/day	7
	Oral prednisone at 1 mg/kg/day	1
Third hospitalization	IV methylprednisolone at 2 mg/kg/day	1
	IV methylprednisolone at 1 mg/kg/day + IV rituximab at 375 mg/m^2^/week	28
	IV methylprednisolone at 0.5 mg/kg/day	6
	Oral prednisone at 0.5 mg/kg/day	9
Third discharge	Oral prednisone at 0.5 mg/kg/day	Until last FU

IV, intravenous; FU, follow-up.

According to Hart and colleagues’ reported data in the Mayo Clinic experience in non-ICI-induced steroid-refractory AIP treatment (15), we decided to administer rituximab (MabThera, Roche) at 375 mg/m^2^ intravenous weekly for 4 weeks in combination with methylprednisolone (1 mg/kg/day), resulting in good clinical and radiologic control. Before rituximab was administered, a blood screening was performed with an immune panel including serum immunoglobulin G4 (IgG4), rheumatoid factor, antinuclear antibodies, anti-mitochondrial antibodies, anti-smooth muscle antibodies, antineutrophil cytoplasmic antibodies, and anti-thyroglobulin antibodies levels, with negative results. Antimicrobic prophylaxis was modified by adding sulfamethoxazole/trimethoprim and acyclovir. Between the third and fourth courses of rituximab, a CT scan described the occurrence of a pancreatic pseudocyst and a dramatic reduction in the peripancreatic fluid. The pseudocyst was treated with the placement of a transgastric prosthesis through endoscopic ultrasound. Serum amylase and lipase levels were recorded during the whole clinical course, and no correlation was shown with the severity of pancreatitis (**Figure 1**). On the following admissions, no finding of pancreatitis was detected on the CT scan (**Figure 2**).

One month after the last rituximab administration, the patient was admitted for fever and a new elevation of inflammation markers, such as serum C-reactive protein and procalcitonin, under antimicrobic prophylaxis. Blood and urine cultures were negative. However, empiric treatment with piperacillin/tazobactam was initiated with a remarkable clinical and laboratory markers’ response. No further episodes followed, and antimicrobic prophylaxis was continued until one year after the last dose of rituximab. On follow-up, there was no clinical or laboratory evidence of pancreatitis. In January 2023, disease progression (PD) was detected in multiple sites (brain, lymph nodes), and second-line chemotherapy was initiated. The brain metastases were treated with whole-brain radiotherapy (WBRT). The patient died of PD in March 2023.

## 3 Discussion

To our knowledge, there are no data on successful steroid-refractory and relapsing ICI-induced pancreatitis treatments. This brief report presents the first case of a patient with atezolizumab-related pancreatitis refractory to steroids successfully treated with rituximab.

IrAEs can affect any organ. Gastrointestinal irAEs, including diarrhea, colitis, and hepatitis, are well documented and studied (1, 2, 19), while cases of pancreatitis after ICIs are reported only in clinical trials and case series (7–10). According to available algorithms, steroids are the mainstay of treatment of most high-grade irAEs. For steroid-refractory cases, recommendations for using immune-modulating agents are extrapolated from the evidence for treating autoimmune diseases (2–4). Rituximab was used to treat steroid-refractory irAEs, such as encephalitis, autoimmune cytopenias, or severe bullous skin disease, with a good clinical and laboratory response (20–22). Considering the similarities with AIP (17), we decided to administer rituximab based on efficacy results from the study of Hart et al. and the international consensus recommendations for AIP treatment (14, 15). The Mayo Clinic experience showed that patients with normal serum IgG4 levels might receive rituximab to manage AIP (15). Furthermore, IgG4 levels were reported to be normal in ICI-related pancreatitis (23) and there were no data on the diagnostic value of IgG4 in ICI-related pancreatitis. Based on this evidence, we were motivated to start a rituximab regimen even though our patient’s serum IgG4 levels were negative. As a result, rituximab showed a surprising clinical and radiologic improvement only 2 weeks after the first infusion, and no relapses were detected during follow-up. Serum amylase and lipase levels were not associated with the clinical trend and did not decrease after steroid therapy, as opposed to previous steroid-responsive ICI-related pancreatitis findings (7, 9, 10). Although elevations in pancreatic enzymes may be helpful in the diagnosis of acute pancreatitis (14, 17, 23), they are not indicated in monitoring response to immunosuppressive treatment based on the resolution of clinical symptoms and radiological abnormalities (17). Serum IgG4 have been described as biomarkers of AIP activity and as predictive markers of relapse (14); however, they were negative in our case. Therefore, we could not identify a possible biomarker for steroid-refractory or relapsed ICI-induced pancreatitis.

Immunosuppression for treating irAEs may increase the risk of opportunistic infections, such as *Aspergillus fumigatus*, *Pneumocystis jirovecii* pneumonia, and cytomegalovirus (2). Therefore, antibiotic prophylaxis should be considered according to available recommendations (2). In our case, sulfamethoxazole/trimethoprim and acyclovir were administered during steroid and rituximab treatment. Only one admission occurred because of a suspected infection. Patients with ongoing steroid therapy should be strictly monitored for the high risk of developing hyperglycemia and/or diabetes (4). Moreover, ICI may cause endocrine pancreatic toxicity (4). Our patient developed diabetes, but we cannot demonstrate whether it is associated with glucocorticoid administration or immune-related adverse events.

The specific mechanism underlying irAEs is still unknown. Atezolizumab is a humanized monoclonal antibody that inhibits PD-L1, a transmembrane glycoprotein expressed on antigen-presenting cells (APCs), including B cells, T cells, macrophages, and dendritic cells (24–26), as well as on nonimmune cells, such as pancreatic islet cells (27). PD-L1 inhibition stimulates the activation of T cells, especially CD8+ cytotoxic T lymphocytes, which are considered the effectors of the anticancer effect of ICIs and are responsible for immune-related toxicities (25, 28). Although the role of B cells and antibodies in irAEs is unclear, their involvement in endocrine disorders, including ICI-related thyroiditis and diabetes, appears to be more evident (25). Considering the onset of diabetes in our patient during immune-mediated pancreatitis, we may assume that there was an immune-mediated injury involving both the exocrine parenchyma and the pancreatic islets, and the efficacy observed with rituximab administration, an anti-CD20 monoclonal antibody acting on B-cell depletion, may indicate an underlying mechanism mainly involving B-cell activity, such as the production of autoantibodies and B- and T-cell interactions (29). Yet, our patient had a negative immune panel screening. Agrawal et al. reported a case of autoimmune pancreatitis and cholangitis induced by nivolumab with normal serum IgG4 levels; the patient underwent surgical resection due to biliary stricture, with IgG4 predominant plasmacytic cell infiltrates on histological examination (30). Considering AIP, its subtypes can be seronegative (17), which may be prevalent in ICI-related pancreatitis (23). Some reasons to explore might be low levels of circulating autoantibodies or the presence of unknown autoantibodies whose mechanism of action has not been described yet.

## 4 Conclusion

Pancreatitis is a rare irAE that responds to steroid therapy in most cases, but data on the management of steroid-refractory pancreatitis are unavailable. We reported the first successful treatment case with rituximab, which can be considered a good and safe option for patients with steroid-refractory, relapsed pancreatitis caused by ICIs and maybe other steroid-resistant IrAEs.

## Data availability statement

The original contributions presented in the study are included in the article/supplementary material. Further inquiries can be directed to the corresponding author.

## Ethics statement

Ethical review and approval was not required for the study on human participants in accordance with the local legislation and institutional requirements. The patients/participants provided their written informed consent to participate in this study.

## Author contributions

Study conception and design: AS and SM. Collection and interpretation of data: AS, SM, and FV. Statistical analysis: N/A. Manuscript drafting: AS, SM, MT, RC, and FV. Manuscript editing: AS, SM, MT, RC, and FV. All authors contributed to the article and approved the submitted version.
